# Clinical audit project in undergraduate medical education curriculum: an assessment validation study

**DOI:** 10.5116/ijme.57da.c89a

**Published:** 2016-09-24

**Authors:** Elina Tor, Carole Steketee, Donna Mak

**Affiliations:** 1 School of Medicine Fremantle, The University of Notre Dame Australia, Fremantle, Western Australia, Australia; 2 Learning and Teaching Office, The University of Notre Dame Australia, Fremantle, Western Australia, Australia

**Keywords:** Assessment in medical education, validity, assessment validation, quality and safety curriculum, population and preventive health curriculum

## Abstract

**Objectives:**

To evaluate the merit of the Clinical Audit Project (CAP) in an assessment program for undergraduate medical education using a systematic assessment validation framework.

**Methods:**

A cross-sectional assessment validation study at one medical school in Western Australia, with retrospective qualitative analysis of the design, development, implementation and outcomes of the CAP, and quantitative analysis of assessment data from four cohorts of medical students (2011- 2014).

**Results:**

The CAP is fit for purpose with clear external and internal alignment to expected medical graduate outcomes. Substantive validity in students’ and examiners’ response processes is ensured through relevant methodological and cognitive processes. Multiple validity features are built-in to the design, planning and implementation process of the CAP.  There is evidence of high internal consistency reliability of CAP scores (Cronbach’s alpha > 0.8) and inter-examiner consistency reliability (intra-class correlation>0.7). Aggregation of CAP scores is psychometrically sound, with high internal consistency indicating one common underlying construct.  Significant but moderate correlations between CAP scores and scores from other assessment modalities indicate validity of extrapolation and alignment between the CAP and the overall target outcomes of medical graduates.  Standard setting, score equating and fair decision rules justify consequential validity of CAP scores interpretation and use.

**Conclusions:**

This study provides evidence demonstrating that the CAP is a meaningful and valid component in the assessment program. This systematic framework of validation can be adopted for all levels of assessment in medical education, from individual assessment modality, to the validation of an assessment program as a whole.

## Introduction

Clinical audit is a cyclical and systematic review of processes, practices and outcomes of healthcare services against clearly defined evidenced-based criteria.^1^ Systematic reviews as such are important in clinical practice, as they place an emphasis on quality improvement in an effort to meet standards and deliver best practice care to patients. Done properly, they provide a robust framework to improve patient care objectively, systematically and in an ongoing fashion.

The population and preventive health curriculum in the undergraduate medical education (MBBS) program in the School of Medicine at the University of Notre Dame Australia (UNDA) culminates in the final year whereby students undertake a supervised capstone project known as the ‘clinical audit project’ (CAP) which is worth 10% of their final mark.^2^ Students are required to identify a SMART (specific, measurable, achievable, related and time-bound) standard relating to healthcare delivery which is published, adopted as health service policy or widely accepted. This is followed by critical appraisal of evidence supporting the chosen standard before assessing whether the healthcare delivered meets this standard.  Students identify and consult with key stakeholders, who are usually staff involved in providing the care being audited, in the planning and dissemination stages of their audit.  As per the normal procedures in clinical settings, students must obtain written approval from the relevant Clinical Quality and Safety Committee (or equivalent) of the health service where they plan to conduct their audit.  To meet the School’s academic requirements, students submit a clinical audit proposal for formative assessment by their peers and examiners (assessment for learning) and a final audit report for summative assessment (assessment of learning) formatted for a medical journal publication.

Validity is possibly the most important consideration in assessment evaluation as it provides confirmation that assessment scores are interpreted and used appropriately and meaningfully.  Contemporary theorists, such as Samuel Messick, Terry J Crooks and colleagues and Michael T Kane, see validity as a unitary or unified concept.^3^^-^^5^  This contemporary view on validity has been adopted as the basis for the revised Standards for Educational and Psychological Testing.^6^^,^^7^  Rather than being viewed as merely a characteristic of an assessment tool or test, validity is more to do with the degree to which the appropriateness, meaningfulness and usefulness of assessment scores permits sound interpretation.  As such, a test or an assessment tool in itself should not be judged merely as valid or invalid. Validity comes into the picture and needs to be addressed when assessment scores are interpreted and used to measure a student’s performance and determine appropriate actions to be taken. That being said, the appropriateness, meaningfulness and usefulness of the interpretation and use of assessment scores are inevitably linked to the more fundamental issue as to whether the assessment instrument is fit for its purpose in the first place. In fact, the process of assuring validity is (or at least should be) a cyclical process that follows the lifecycle of the design, development, implementation and evaluation phases of every assessment.   

According to Samuel Messick, there are two main overarching categories of threats to validity of assessment scores, namely construct irrelevant variance (CIV) and construct under representation (CUR).^3^^,^^8^  CIV is a threat to validity due to uncontrolled extraneous variables which may impact on students’ performances in an assessment, and which consequently affects the accuracy of results and the legitimacy of subsequent decisions made based on those results.  CUR, on the other hand, occurs when what is assessed does not reflect the relevant knowledge, skills or attitudes, which compromises the meaningful and appropriate interpretation and use of the resulting scores. 

The recent call for a unified view on validity is imperative and timely as it highlights the fact that this construct exists on a continuum, as opposed to the commonly assumed binary notion of ‘valid’ or ‘not valid’.^3^^,^^5^^,^^8^^,^^9^ Often, evaluation practices focus solely on reliability and overlook the more complicated question of validity, which, unlike reliability, cannot be measured numerically. Instead, assessment validation involves a process of investigation and data collection to identify the evidence required for rebuttal against significant threats to validity at every stage of the assessment cycle.  Contemporary theorists therefore emphasize the importance of an assessment validation study, and that validity in assessment score interpretation and use should be approached as a hypothesis, rather than be assumed.^3^

 This validation study is an answer to the aforementioned call for a holistic and unified view of validity and assessment validation research. It is also one of the many parallel initiatives on the part of UNDA’s medical school, for continuous improvement and refinement of each and every assessment components - a concerted effort towards a more defensible, meaningful and fair assessment program. 

This paper describes the implementation and findings from the aforementioned assessment validation study conducted to determine the extent to which the CAP (and the interpretation and use of the resulting assessment scores) is aligned to the MBBS curriculum outcomes and meets its purpose of ensuring that UNDA medical graduates have the ability to design and implement a clinical audit in accordance with requirements of a health service’s clinical quality and safety committee. Using a contemporary assessment validation framework^3^^-^^5^^,^^8^^,^^9^  this study reframes validity as a set of questions and associated validity criteria that were used to determine whether students’ scores in the CAP assessment genuinely reflect attainment of learning outcomes, and  ultimately, that the CAP is a  relevant and meaningful learning experience for students.

## Method

### Study design

This was a cross-sectional assessment validation study at one medical school in Western Australia.

### Participants and data collection

The study involved retrospective qualitative analysis of the design, development, implementation and outcomes of CAP, through conceptual analysis, document analysis and audit of processes.  This is complemented by retrospective quantitative analysis of CAP assessment data from 4 cohorts (2011- 2014) of final year medical students (N ± 100 each cohort) for psychometric properties of CAP scores.

### Procedure

Consistent with the process of educational assessment development, the process of validation begins with the end in mind.^3^ As such, an interpretive argument was first  formulated (Table 1) consistent with Kane’s argumentative approach to assessment validation.^5^ This interpretive argument is similar in function to a hypothesis in other types of research, from which associated validation (or research) questions were derived.  This provides a pathway for interpreting assertions about the purpose of an assessment and how resulting scores should be construed.^5^ The interpretive argument (Table 1) outlines the purpose of the CAP and its relevance to student learning according to the target construct, the target domain and the target sub-domain. Breaking the interpretive argument down into these component parts allows for a more focussed validity arguments to be developed.

The interpretive argument outlined above, although specified according to the relevant component parts, were still relatively broad statements that required further unpacking in order to examine validity at a more granular level.  Therefore, Crooks, Kane and Cohen’s chain model of educational assessment^4^ was used to frame specific validation question (VQ) at each stage of the CAP:

**VQ1.** Is the clinical audit project fit for purpose (design and planning link)?

**VQ2.** Are the methodological and cognitive processes involved in the clinical audit project relevant (design and planning link)?

**VQ3.** Are the examiners’ judgments about a student’s performance really reflect the student’s ability in the domain assessed (scoring link)?

**VQ4.** Are the clinical audit scores generalizable to all possible scores across clinical audit topics and all examiners who have scored the reports (generalization link)?

**VQ5.** Are the aggregate total scores for clinical audit reports assessing one common underlying construct? Are there possibilities of construct under-representations (aggregation link)?

**VQ6.** Are there sufficient conceptual and empirical evidence to support the extrapolation from the target domain assessed via the CAP to the target construct, i.e. the overall competence of a safe medical graduate (extrapolation link)?

**VQ7.**  Is the evaluation of scores based on sufficient assessment information including the limitation arising from measurement errors (evaluation link)?

**VQ8.** Is there a clear, fair, explicit and well-communicated decision rule as a basis for important decisions based on clinical audit scores (decision link)?

**VQ9.**  What are the educational utilities and impacts of the clinical audit project? Is there evidence of unintended consequences? What are the social consequences of students doing the clinical audit project (impact link)? 

One powerful message from Crooks, Kane and Cohen’s chain model of educational assessment is that there are a series of interrelated stages in all assessment practices, and, threats to validity can happen due to practices at every link in an assessment cycle. If any one of these links are weak or indeed broken, then this affects the overall integrity of the assessment scores. As such, it is crucial that assessment developers identify practices with plausible threats to validity, that is, the weakest link so that attempts can be made to have quality assurance mechanism in place to mitigate these threats and to strengthen overall integrity.

Guided by these validation questions, plausible validity threats were identified, which allowed multiple sources of theoretical and scientific evidence to be identified and subsequently collected, collated and documented – all of which are paramount to justify the meaningful and proper interpretation of assessment scores from the CAP.  Appendix 1 provides a comprehensive overview of the methodological framework used to systematically guide the validation study for the CAP.

Ethics approval was not required as this validation study was conducted as part of a quality improvement review of the design and implementation of the CAP.

**Table 1:** Basic structure for interpretive arguments: purpose, intended scores interpretation and use, target construct, target domain and target sub-domain

Purpose of clinical audit project		
· The CAP is a capstone project for students to synthesise what they have learned from the population and preventive health curriculum (includes evidence based medicine, research, health systems, quality and safety, and professionalism) in the first three years of the MBBS and apply it in a real-life clinical workplace to measure and advocate for improvements in the quality of an aspect of patient care.		
Scores interpretation and use		
· CAP scores contribute to 10% of student’s overall final year grade		
· CAP scores are used to identify students who have demonstrated significant deficiency in their ability to conduct and report a CAP, so that these students can undergo a remediation program, either to improve on the existing audit project or conduct a new project		
Target construct	Target domain	Target sub-domain
The target construct in the assessment program for final year students in MBBS course is the overarching curriculum outcome, i.e. the competence of a safe medical practitioner.	The target domain assessed in the CAP is the competency in conducting a real clinical audit project, reporting the results, disseminating the findings and reflecting on the experience.	The task of conducting, reporting, disseminating findings and reflecting on related experience are guided by documented steps (or sub-domains), which include the Identification of a topic for audit (rationale and significance); identification of a SMART standard for audit in the a clinical setting; Appropriate methodology; Appropriate data analysis; Appropriate reporting of findings; Reflection; Appropriate involvement of stakeholders.

## Results 

Data, which comprised evidence collected against each stage of the assessment practices in the CAP cycle, were analysed and evaluated to determine whether sufficient evidence existed for rebuttal against potential threats to validity of CAP scores in each stage of the CAP. Findings are reported against each of the validation questions which guided the analysis in each of the CAP assessment links.

### Fit for purpose (VQ1)

The first phase of validation (and question) relates to the most fundamental and overarching aspect of validity arguments - whether there is any evidence to show that the CAP is fit for its purpose in the MBBS curriculum. 

In real world clinical practice, clinical audit is a part of a health service’s quality plan to assure competency, for the ultimate goal of improving the outcome and quality of patient care.^10^ Research shows that the practice of clinical audits result in improvements in care provided by medical practitioners.^11^  Clinicians undertaking clinical audits benefit professionally from systematic examination of their clinical practice and ongoing education, and their patients benefit from improved care. 

Recognising that good habits should start early, the Australian Medical Council (AMC) specifically states that an undergraduate medical student should develop:

“the abilities and disposition to self-evaluate their own professional practice; demonstrate lifelong learning behaviours and fundamental skills in educating colleagues. Recognise the limits of their own expertise and involve other professionals as needed to contribute to patient care.”^12^ 

The CAP has been developed with this goal in mind. Since 2008, it has been embedded in the MBBS program at UNDA as a means of helping students develop the knowledge, skills and attitudes required to systematically reflect, measure and improve some aspect of patient care in the workplace. By designing and conducting the CAP, and reporting it to their supervisors, students develop the reusable skills of reflection and enquiry to explore future issues upon graduation and into their professional careers.

### Relevant methodological and cognitive processes (VQ2)

The second phase of validation relates to whether the cognitive and methodological processes involved in conducting a CAP are relevant to the graduating students in their future practice of medicine.  Firstly, the relevance of methodological and cognitive processes involved in the CAP is supported by theories on professional competence.  The literature on change in medical practitioners’ behaviours suggests that identification and awareness about the size of the gap between actual and desired practice, as stipulated in professional practice guidelines, standards or benchmarks, influences the motivation for change and learning.^13^  Clinical audit is therefore a natural and affirmative route to quality assurance in medical practice, either as a system wide measure, or ideally, proactively pursued at the individual practitioner’s level.

Secondly, the competency to conduct a clinical audit project is in fact a lifelong learning skill essential for ongoing professional expertise development and improvement in quality of service and care to patients. Professional colleges are increasingly requiring members to conduct clinical audits as part of their ongoing quality assurance activities. Ongoing reflection on the standard of care provided to patients is required to achieve the best health outcomes. Students need to learn about this concept and know how to implement it in practice.  Early first-hand exposure to the process of conducting a clinical audit gives UNDA graduates a head-start as most Australian and international medical colleges require their fellows and trainees to conduct clinical audits for ongoing professional learning, quality improvement and as part of their professional accreditation and registration. 

In addition, the detailed marking rubrics for the CAP provide examiners with operational definitions of the intended methodological and cognitive processes involved in clinical audit.  They are developed (and revised) based on established frameworks for clinical audits used in health departments across Australia and New Zealand.  Consequently, there is a direct alignment between the intended methodological and cognitive processes in the CAP and best practice guidelines for clinical audit in the real clinical world. This detailed marking rubrics, and the comprehensive Clinical Audit Project Handbook, scaffold students’ learning of the skills, knowledge and competence in designing, planning, implementing a real clinical audit and reporting of the findings.

The CAP also represents a tangible expression of ‘service’ or ‘socially accountable’ element of the UNDA’s mission that students should give back to the community that supports and enables their learning.

### Validity in examiner’s inferences (VQ3)

Scores are awarded for the CAP based on the examiners’ review of individual students’ clinical audit reports.  This is the first level of inference that needs justification to ensure validity in the CAP scores. 

The first support for the validity of examiners’ response process in scoring is in the use of a sufficiently detailed marking rubrics.  This marking rubrics help to structure observations and guide examiners on the key evidence to look for in students’ project reports, in order to make informed judgments about the quality of performance.  This is one of the fundamental pre-emptive measures to control for construct irrelevant variance (CIV) due to variability in scoring between examiners. It is also the substantive validity of scores which according to Samuel Messick,  supports the inferences made by examiners, in translating their observation of student’s CAP report to a score awarded for each piece of student’s CAP work.^3^^,^^8^

Standardization of intended cognitive processes and tasks in the form of a detailed marking rubrics also serve to facilitate discussion about the properties of assessment procedures between student/student, and student/teacher.  It is, therefore, a sustainable assessment because it helps create reflexive learners with skills to make informed judgements about their own performance.^14^ 

Secondly, the validity of individual examiners’ response processes in scoring (inferences from observation to scores) is also enhanced by the use of a global score scale in the marking scheme, in addition to the criterion-referenced numerical rating scales, which can be somewhat reductionist when used alone.  The use of a global score scale is a deliberate effort to capture and emphasize the integrative nature of competence. It is to ensure that authentic and holistic judgement and assessment is not overlooked by both students and examiners.

The scores and performance in the CAP, therefore, carry more meaning and value than simply the aggregation of scores from the criteria specified in the marking rubrics.  More importantly, it captures the quality of the whole CAP, particularly on how well each of the individual methodological procedures and cognitive processes are interwoven to produce the intended outcomes on the quality of the particular clinical practice being audited.  This is the aspect of validity which can easily be compromised in the pursuit of criterion-referencing, objectivity, and reliability in assessment practices.

### Generalisability of Scores (VQ4)

Variability between clinical audit topics as well as examiners may compromise the generalizability, or, inferences from the observed scores (i.e. scores obtained by student in a clinical topic, assessed by a particular examiner) to the universe scores (i.e. all possible scores if a different clinical audit topic was selected and/or a different examiner has marked the report). At the School of Medicine at UNDA, the following mechanisms are in place to minimise the threats to generalizability of clinical audit scores:

### Tightening universe of generalisation

As students are allowed to choose their own topics for their CAP, the topics chosen may vary greatly in terms of methodological and cognitive demand, hence contributing to CIV in their scores. To control for possible effects of variation in difficulty of tasks due to differences in complexity between different clinical audit topics, students are provided with a list of clinical audit topics.  These topics are provided by supervising clinicians or by the health service’s clinical quality and safety committees.  The clinical audit project’s academic coordinator assesses the suitability of these topics for medical students’ CAP and removed unsuitable topics before presenting them to students.

### Standardization in assessment criteria

In addition to the above, the assessment criteria and rubrics for CAP are designed to target the generic overarching methodological and cognitive processes which are applicable across most, if not all, clinical audit topics. This also helps in the generalizability of scores across topics, reducing the threats of CIV due to topics chosen for the CAP.

### Standardization in response process in marking

Variability in examiners' scoring, on the other hand, is controlled through the provision of detailed marking rubrics with descriptors and numerical rating scales for each distinct criterion of assessment.  The examiners (N=3 to 4) involved also meet at least twice a year (before commencing the formative and summative assessment of students’ CAP work) to discuss the interpretation and use of each marking criteria and rubric. CAP proposals and reports by students from previous cohorts are used as the basis for initial calibration of judgement on performance standard between examiners, particular for examiners who are new to the role.

In addition to the calibration of judgment on performance standard between examiners, a marking moderation exercise is conducted based on 5 to10% of audit reports marked by all examiners.  Detailed analysis outcomes on scoring consistency between individual examiners are discussed and discrepancies in scoring consistency (if there are any) are resolved before each examiner embarks on the marking of the remaining 90 to 95% of audit reports. 

As a result of these quality assurance mechanisms, over the last four years, a highly satisfactory level of inter-rater consistency reliability has been achieved, which implies the results are generalizable across examiners (Table 2).

**Table 2:** Inter-examiner consistency reliability of Clinical Audit Project scores

Year	Number of Examiner Involved	Pair Intraclass Correlation Coefficient (ICC) (2,2)	Intraclass Correlation Coefficient (ICC) All Examiners (3 or 4) (2,3) or (2, 4)
		Two way-mixed for 2 examiners	Two way-mixed for 3 or 4 examiners
2011	3	0.878 - 0.891	0.891
2012	3	0.891 - 0.893	0.936
2013	3	0.967 - 0.999	0.969
2014	4	0.740 - 0.870	0.800

### Validity in aggregate scores (VQ5)

The scores from each criterion and sub-sections of the CAP marking rubrics are aggregated to give a total score.  This total score is taken to be the measure for competency in conducting and reporting a clinical audit - the target domain. Hence, this involves inference from the universe scores (from all possible aspects of clinical audit methodology and cognitive processes) to the target domain (i.e. competency in conducting a real clinical audit and reporting and reflecting on the findings). 

The validity of the aggregated CAP score is supported conceptually by the substantive validity of the marking rubric itself (as discussed in the preceding section on the substantive validity of response processes for students and examiners), i.e. the alignment of the marking rubric with existing best practice guidelines for clinical audit used by various departments of health across jurisdictions in Australia and New Zealand.

In addition, the validity of the aggregated scores is supported empirically by the scores that have demonstrated highly satisfactory internal consistency reliability (Table 3). The internal consistency reliability coefficient (i.e. the Cronbach's alpha coefficient) based on scores from the CAP scale have been above 0.8 between all individual items on the scale; and above 0.7 between scores from main sections of the scale.  This indicates that the clinical audit assessment scale is indeed measuring a common underlying construct which has been conceptually operationalised as the competency in conducting and reporting a clinical audit project.

**Table 3:** Internal consistency reliability of Clinical Audit Project scores

Academic year	Internal consistency reliability Cronbach's alpha overall score scale	Internal consistency reliability Cronbach's alpha between section scores
2011 (N=99)	0.82	0.72
2012 (N=104)	0.90	0.80
2013 (N=98)	0.85	0.75
2014 (N=95)	0.85	0.74

### Validity of extrapolation (VQ6)

The CAP is one component of the assessment program in the final year of the MBBS course, a year-long integrated curriculum.  As such, the CAP score is aggregated with scores from all other assessment modalities to become the overall score for final year.  This involves extrapolation whereby inference is made from the target domain assessed (i.e. competency in conducting and reporting a real clinical audit) to the target construct (i.e., overall clinical competence of safe medical graduates).

Conceptually, the alignment between the target domain assessed (i.e. competency in conducting and reporting a clinical audit project) and the target construct (i.e. the overall competence of safe medical graduates) is described in validation question 1.  In addition, the authenticity of tasks involved in the CAP, which includes authenticity in physical context, social context, resources and reports, also supports the validity of extrapolation or transferability of target competency assessed in CAP to the real clinical workplace upon graduation. Empirically, the validity of extrapolation is supported by the correlations between clinical audit scores and scores from other assessment modalities in the MBBS assessment program for final year students.

As shown in Table 4, the bivariate correlation analysis shows students’ CAP scores are, in most instances, significantly and moderately correlated with their scores in written exams, workplace-based assessments, objective structured clinical examinations (OSCE), and personal and professional development portfolios. These empirical correlations indicate that the CAP is assessing a distinct domain of a common underlying construct, namely clinical competence as required to be safe medical graduates who can assess and develop strategies to improve the quality of patient care, which is somewhat different from the other domains of competence as assessed in other summative assessment components.

**Table 4:** Correlation between Clinical Audit Project scores and scores from other assessment modalities†

Year	Written exam	Workplace-based continuous assessment	Objective structured clinical examination	Personal professional development portfolio
2011	0.283**	0.245**	0.211**	0.132*
2012	0.367**	0.283**	0.275**	0.328**
2013	0.280**	0.212**	0.120	0.040
2014	0.310**	0.409**	0.317**	0.074

†Bivariate correlation between clinical audit scores with scores from all other summative assessment components which are aggregated with clinical audit scores to be the overall MBBS final year scores. *Correlation is significant at the 0.05 level (2-tailed) **Correlation is significant at the 0.01 level (2-tailed)

### Validity in evaluation of scores (VQ7)

The validity of evaluation and interpretation of the CAP scores is ensured through a multi-disciplinary team consisting of public health physicians, educationalists and an applied psychometrician working together in the implementation of the CAP.  Assessment information, particularly on the psychometric properties of scores, are constantly monitored including limitations in terms of measurement errors.  This information is communicated to all involved in evaluation and decision making based on the clinical audit scores.  Before the clinical audit scores are evaluated, there is a standard setting mechanism in place to determine the minimum acceptable performance standard, that is, the pass/fail cut score on the CAP assessment scale.  The standard set pass mark (or pass/fail cut score) is the minimum expected standard reference point for the CAP task performance. 

The final grading (judgement and evaluation) of the CAP is done using a different scale - the common standardized score scale used across all Schools at UNDA, where the pass mark is fixed at 50 (and with a minimum of zero and a maximum of 100). Therefore, an additional process of scaling or score equating is necessary, whereby the pass/fail cut score is used as an anchor to scale (or equate) the raw scores from the clinical audit assessment scale to scores on the common standardized UNDA score scale. 

**Figure 1:**
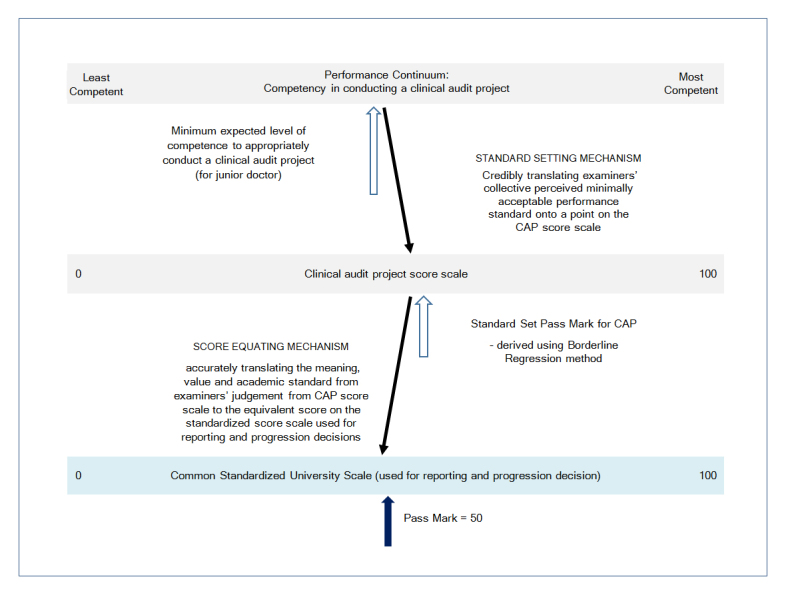
Validity in evaluation of CAP scores - standard setting and score equating mechanism

Through the standard setting and score-equating processes, the meaning, value, and academic standard of the CAP scores is linked and translated accurately across from CAP score scale to the final scores on the University standardized score scale, for reporting and for further aggregation with scores from other assessment components (Figure 1). This is particularly important because CAP scores are aggregated (with a 10% weighting) with scores from other assessment modalities to give the total scores for the final year MBBS unit. 

### Decision validity (VQ8)

The decision rule for CAP results (in keeping with the decision rules for the assessment program) is criterion and standard-referenced.  Performance standard is evaluated based on the standard set pass mark, with the standard error of measurement being used as quantitative qualifier in the decision rule.  Students with a CAP score of more than two standard error of measurement below the standard set pass mark, are subject to one other independent scoring by a different examiner.  If the second examiner’s mark is also more than two standard error of measurement below the standard set pass mark, an appropriate remediation program will be discussed and determined by the academic coordinator for CAP and the Dean.  The student will be offered the opportunity to resubmit the CAP report based on the specific comments outlined by the examiners, either based on the existing CAP or to work on a new project, in which case the student will be closely supervised by a faculty member. The decision making based on CAP scores therefore encourages students to learn and achieve the intended learning outcomes, rather than being simply punitive.

### Impact validity (VQ9)

#### Educational utility

The CAP serves multiple purposes in the MBBS curriculum. Its design ensures that it is a constructive learning experience for students and constitutes ‘assessment as learning’, ‘assessment for learning’ as well as ‘assessment of learning’. In recognition of Biggs and Tang's assertion that 'assessment drives learning', the design of the CAP drives students toward attaining the intended learning outcomes through active engagement in the process of assessment.^15^ As a capstone project, the CAP experiences build on students’ prior learning in the first three years of the Population and Preventative Health curriculum, culminating in the synthesis and application of knowledge and technical skills in a real life clinical setting, based on a real life topic affecting patient outcomes. In addition to a comprehensive CAP handbook, students’ learning is scaffolded by various learning activities, such as workshops, seminars and lectures, which are distributed throughout the year.

Through the multi-perspectives feedback processes of self-assessment, peer-assessment, lecturers’ formative assessment and feedback provided on students’ CAP proposals, both lecturers and students are actively engaged in dialogue about academic standards. It is through these processes of continuous dialogue that assessment standards are socially constructed to enable an atmosphere of mutual trust be established between lecturers and students for learning to take place.^16^  

#### Consequential validity

There is strong evidence to support the validity of decision rules for CAP scores (also reported under Validation Question 7).

A student whose score lies more than two standard error of measurement below the pass mark is deemed to be of concern with regards to competency in clinical audit.  These students will be given the opportunity to undergo an intensive focussed remediation with a faculty member during their elective rotation, which is scheduled after the final summative assessments, but before the final Board of Examiners which authorises graduation. This process ensures that decisions made based on CAP scores are to support further learning and are not simply punitive. The validity of decision rules for CAP scores also contribute to the consequential validity, that is, defensibility and fairness of decisions based on clinical audit scores.

#### Practicality and acceptability

The CAP requires an estimated average of four hours of student engagement per academic week or 120 hours per academic year and contributes 10% towards the students’ overall summative assessment results.  Students are advised to collect and analyse data on not more than five demographic (exposure) variables and up to 10 outcome variables from 20 to 50 patients.  They are briefed very clearly that data collection should not require interaction with patients after the patient’s discharge from the health service.  Students, therefore, understand and accept the CAP as a practical component of the curriculum and assessment modality.

#### Social impact

The CAP program is well-received by the health services involved. Some health services even post flyers to recruit UNDA medical students to conduct clinical audits in their institutions on areas requiring investigation. Every year, many clinical audit topics that constitute ‘priority areas’ are identified by individual clinicians and/or the health service for students to choose from.  There are also evidence of real changes happening in clinical practices as a result of the outcomes from student’s clinical audit projects and reports following dissemination to the governing bodies at the respective health services.

The impact of the CAP on UNDA graduates after they enter the medical workforce is currently being studied. Preliminary findings indicate that the CAP’s influence continues post-graduation and seems to be instilling the ethos of quality improvement in UNDA graduates.  These graduates have reported that the CAP equipped them to participate in and initiate quality improvement activities in their workplaces, and to provide and advocate for best practice, evidence-based care for their patients. They also reported that CAP improved their competitiveness when applying for jobs and specialist training positions.^17^

## Discussion

The evidence from the conceptual analysis on the content relevance of the CAP presented against validation questions 1 and 2, strongly suggests that the CAP is fit for purpose and is a valid and meaningful component of the assessment program for final year medical students. There is external and internal alignment between the curriculum outcomes suggesting that the CAP is meeting professional accreditation standards in preparing students for practice upon graduation.

Further validity arguments as presented against validation questions two to eight also demonstrate sufficient theoretical rationale and empirical evidence to support the adequacy and appropriateness of inferences and decisions made based on the CAP scores.

In undertaking this systematic validation study underpinned by a holistic and unified view of validity, curriculum and assessment developers have been intimately involved in reviewing and questioning their practices in the design, development and implementation of the CAP.  This assessment validation framework has simultaneously acted as robust quality assurance framework whereby checks undertaken throughout the lifecycle of the CAP have resulted in improvements and a more systematic approach to the overall CAP design.  The principle of ‘begin with the end in mind’ has been adopted at every stage in the assessment cycle and it is through this deliberate effort that many pre-emptive quality assurance measures have subsequently been built-in as inherent validity features ‘by design’.^18^  For example, since the inception of the clinical audit in 2008, this systematic approach to design has progressively resulted in the implementation of construct mapping, the development of the clinical audit handbook and the marking rubrics, the introduction of scoring moderation, scaling of raw scores and refining of the rules for student progression. This simultaneous validation / quality assurance process also enables post-hoc monitoring of potential anomalies in the CAP scores, thus opening up opportunities for further investigation and action to be taken (if necessary) before final decisions and judgements are made. An indirect outcome of this is greater staff and student confidence in the CAP as an assessment component and in the capacity to judge student performance in this area of the curriculum.

It is important to reiterate that the quality of any assessment program is dependent on the quality of the combination of each and every one of its building blocks and not in the superiority of any one of them alone.^19^^,^^20^ The quality of an individual assessment component, however, its alignment and contribution to the common goal of the assessment, is crucial to ensure the integrity of the assessment program.  Therefore, a validation study for an individual assessment component is crucial, as well as the validation study for the assessment program as a whole.

## Conclusions

This validation study is by no means a full-scale Rolls-Royce type of validation study as in the case of the public examinations in England.^21^ Rather, it is the intention of the authors, which has been pioneered and showcased through this study, to present a practical, workable assessment validation framework for medical education, that is underpinned by contemporary validity theories and models of educational assessment measurement. This systematic framework of validation can be adopted for all levels of assessment in medical education, from individual assessment modality to the validation of an assessment program as a whole.  It is also important to note that the application of this framework of assessment validation requires ongoing commitment, not only from the curriculum and assessment developer and administrators, but also from policy makers, to ensure validation is seen as part of the fabric of all assessment initiatives.  This is necessary to improve the haphazard processes of assessment operating in many educational institutions in general and medical education in particular, to ensure all assessment initiatives actually contribute positively in achieving the overall curricular goal or outcomes.

### Conflict of Interest

The authors declare that they have no conflict of interest.

## Supplementary material

Supplementary file 1 Appendix 1. Methodological framework for clinical audit project scores validation study
