# Supplementary material for: Clinical audit project in undergraduate medical education curriculum: an assessment validation study
Source: Int J Med Educ. 2016 Sep 24;7:309–19. doi: 10.5116/ijme.57da.c89a (PMC5056027; doi:10.5116/ijme.57da.c89a)
Supplement: Supplementary file 1 — Appendix 1. Methodological framework for clinical audit project scores validation study [file ijme-7-309-S1.pdf]

Appendix 1.

Methodological framework for clinical audit project scores validation study

**Stages in the chain of assessment cycle**	**Inferences/assumptions in need of close scrutiny in validation**	**Potential threats to validity**	**Methodology of validation**
Design phase of CAP<br>**VQ1**	Clinical audit project (CAP) is fit for purpose, i.e. it is aligned with MBBS curriculum outcome statements and externally aligned with AMC Graduate Outcome statements;<br>CAP project is underpinned by sound educational and lifelong learning principles for medical practitioners;	Misalignment	- Document analysis to map clinical audit project to learning outcomes in MBBS curriculum and AMC graduate outcome statements<br>- Conceptual analysis; - linking and documenting theoretical foundation for clinical audit and its relevance to lifelong learning and development of medical professionals
Planning and development phase for CAP in the curriculum and assessment program<br>**VQ2**	1. Students provided with sufficient information about the CAP; they are well motivated; working conditions are fair for all students;<br>2. Sufficient constructive scaffolding activities for students;<br>3. Practicality of CAP project – health services and CQSCs need empirical evidence for service quality improvement	1. Construct-irrelevant variance (CIV) due to lack of information;<br>2. CIV - Lack of consistent scaffolding<br>3. Unintended consequences	Audit /document analysis of CAP handbook; CAP program timeline (2013-2014)<br>Clarification of how CAP relates to outcomes that are authentic/relevant to clinical practice<br>Analysis of feedback from student surveys; alumni feedback; stakeholder feedback, which is used to modify CAP tasks for future cohorts
Scoring of CAP reports<br>**VQ3**	1. Examiner's judgments (claims) about student's performance genuinely reflect students' ability in the task/domain assessed;<br>2. Scoring process is valid – examiner's response process in observing/reading student's CAP report and scoring is based on a valid scoring rubric;	1. CIV in scoring process	- Audit of scoring process. Collaborative design of scoring rubric to ensure it aligns with outcomes.<br>- Empirical analysis of scores derived from the scoring instrument for internal consistency; inter- rater reliability;<br>- Conceptual/Document analysis (scoring rubric)
	3. Scoring rubrics/scales sufficiently capture the most important qualities of student performance, i.e. the targeted cognitive and methodological processes in CA in real clinical practice;	2. Construct under representation (CUV) in scoring rubric	
Generalisation of CAP scores<br>**VQ4**	1.Scores for each criterion in the assessment rubric are consistent across different audit topics chosen;<br>2. Each scoring criterion in the rubric is applicable to a variety of clinical audit topic;	- CIV due to clinical audit topic chosen;	- Document analysis and expert opinion - the homogeneity of the assessed domain; the number of tasks required for adequate generalizability;<br>- Document analysis/audit of CAP planning and development process<br>- Empirical analysis of CAP scores – for internal consistency between section scores
	3. Scores across different tasks assessed (within the scoring rubrics) are consistent and correlated;<br>4. The criteria assessed in a CAP report covers sufficiently the scope of the project as applied in real clinical setting;	- CIV due to low inter-task correlation;<br>- CUV due to too few tasks included as assessed criteria;	
Aggregation of CAP scores<br>**VQ5**	1. Aggregation of scores from individual scoring items to be the overall CAP project score is valid;<br>2. Aggregation of sub-section scores from the formative assessment of student's CAP proposal is meaningful for student in guiding their next step in learning on clinical audit project	- CUV	- Empirical analysis of scores – internal consistency reliability of overall scores; subsection scores

**Stages in the chain of assessment cycle**	**Inferences/assumptions in need of close scrutiny in validation**	**Potential threats to validity**	**Methodology of validation**
Extrapolation of CAP scores– CA scores aggregated with marks from all other assessment components<br>**VQ6**	Extrapolation from CAP scores to overall competence as a safe intern (the target domain) is valid<br><br>The scores in CAP is a predictor of performance in the clinical practice workplace upon graduation	- Inconsistency in internal structure between CAP scores and scores from all assessment modalities	1. Empirical analysis of clinical audit score data for the following:<br>a. internal consistency between CAP scores and scores from all summative assessment components forming part of the overall aggregate score for fourth year students;<br>b. Correlation between CAP scores and scores from all other summative assessment components;<br>2. Conceptual analysis - alignment between competency in CAP and overall competence as intern
Interpretation of CAP Scores<br>**VQ7**	CAP scores are evaluated with reference to the standard set pass mark and standard error of measurement (SEM) which is determined credibly	- CIV due to the meaning and value of scores failed to be translated accurately to the final scores used for final evaluation / judgement;<br>- Inadequately supported construct language used in scores interpretation	- Document analysis and decision making process audit:
Decision & Actions based on CAP scores – **VQ8**<br>*CAP scores are evaluated to identify students who have demonstrated serious deficiency*	Decision made based on CAP scores is underpinned by an explicit decision rules, explained to examiners and students	CIV	- Document analysis – decision rules; form of reporting or feedback to be used
Consequences /Educational Impact – **VQ9**	CAP brings more positive educational impact and non-significant unintended consequences	Unintended consequences	Analysis of feedback from student surveys; alumni feedback; stakeholder feedback
